# Artificial Intelligence–Assisted Capsule Endoscopy Versus Conventional Capsule Endoscopy for Detection of Small Bowel Lesions: A Systematic Review and Meta‐Analysis

**DOI:** 10.1111/jgh.16931

**Published:** 2025-03-13

**Authors:** Arkadeep Dhali, Vincent Kipkorir, Rick Maity, Bahadar S. Srichawla, Jyotirmoy Biswas, Roger B. Rathna, Hareesha Rishab Bharadwaj, Ibsen Ongidi, Talha Chaudhry, Gisore Morara, Maryann Waithaka, Clinton Rugut, Miheso Lemashon, Isaac Cheruiyot, Daniel Ojuka, Sukanta Ray, Gopal Krishna Dhali

**Affiliations:** ^1^ Academic Unit of Gastroenterology Sheffield Teaching Hospitals NHS Foundation Trust Sheffield UK; ^2^ School of Medicine and Population Health University of Sheffield Sheffield UK; ^3^ Faculty of Health Sciences University of Nairobi Nairobi Kenya; ^4^ Institute of Post Graduate Medical Education and Research Kolkata India; ^5^ University of Massachusetts Chan Medical School Worcester Massachusetts USA; ^6^ College of Medicine and Sagore Dutta Hospital Kolkata India; ^7^ University Hospitals of Leicester NHS Trust Leicester UK; ^8^ Faculty of Biology Medicine and Health The University of Manchester Manchester UK

**Keywords:** artificial intelligence, bowel, capsule endoscopy, diagnosis, small intestine

## Abstract

**Background:**

Capsule endoscopy (CE) is a valuable tool used in the diagnosis of small intestinal lesions. The study aims to systematically review the literature and provide a meta‐analysis of the diagnostic accuracy, specificity, sensitivity, and negative and positive predictive values of AI‐assisted ce in the diagnosis of small bowel lesions in comparison to ce.

**Methods:**

Literature searches were performed through PubMed, SCOPUS, and EMBASE to identify studies eligible for inclusion. All publications up to 24 November 2024 were included. Original articles (including observational studies and randomized control trials), systematic reviews, meta‐analyses, and case series reporting outcomes on AI‐assisted ce in the diagnosis of small bowel lesions were included. The extracted data were pooled, and a meta‐analysis was performed for the appropriate variables, considering the clinical and methodological heterogeneity among the included studies. Comprehensive Meta‐Analysis v4.0 (Biostat Inc.) was used for the analysis of the data.

**Results:**

A total of 14 studies were included in the present study. The mean age of participants across the studies was 54.3 years (SD 17.7), with 55.4% men and 44.6% women. The pooled accuracy for conventional ce was 0.966 (95% CI: 0.925–0.988), whereas for AI‐assisted ce, it was 0.9185 (95% CI: 0.9138–0.9233). Conventional ce exhibited a pooled sensitivity of 0.860 (95% CI: 0.786–0.934) compared with AI‐assisted ce at 0.9239 (95% CI: 0.8648–0.9870). The positive predictive value for conventional ce was 0.982 (95% CI: 0.976–0.987), whereas AI‐assisted ce had a PPV of 0.8928 (95% CI: 0.7554–0.999). The pooled specificity for conventional ce was 0.998 (95% CI: 0.996–0.999) compared with 0.5367 (95% CI: 0.5244–0.5492) for AI‐assisted ce. Negative predictive values were higher in AI‐assisted ce at 0.9425 (95% CI: 0.9389–0.9462) versus 0.760 (95% CI: 0.577–0.943) for conventional ce.

**Conclusion:**

AI‐assisted ce displays superior diagnostic accuracy, sensitivity, and positive predictive values albeit the lower pooled specificity in comparison with conventional ce. Its use would ensure accurate detection of small bowel lesions and further enhance their management.

## 1 Introduction

Radiological evaluation of the small bowel has in the recent past been a great challenge owing to the limitation in evaluation of depth and diagnostic yield offered by former techniques such as the push enteroscopy [1]. With the diagnostic restrictions brought about by the anatomical configuration of the small intestines, professionals resorted to intraoperative enteroscopy for complete evaluation, although it is associated with morbidity and mortalities in up to 17% and 5% cases, respectively [2]. This was the case until the early 21^st^ century, which came along with the advent of capsule endoscopy (CE) with diagnostic yields of up to 87% in acute gastrointestinal bleeding [3]. Small bowel capsule endoscopy (SBCE) has since then been the first‐line modality for the diagnosis of several small intestinal diseases such as small bowel tumors, Crohn's disease, polyposis syndrome, Celiac disease, and suspected small bowel bleeding [4, 5]. SBCE entails ingestion of a capsule that then transmits images at a rate of two to six frames per second in 8–12 h depending on the battery life. Thousands of 512‐by‐512‐pixel high‐resolution images, transcribed into a video, are then generated and interpreted by a clinician taking up to an hour per video [6, 7].

Although SBCE offered such a reprieve from the challenges of the former diagnostic techniques, its effectiveness was, however, limited by factors such as poor battery life, and long reading times considering the number of images needed to be taken [5, 8]. Technological advancements have largely sorted out the battery life issues, leaving the reading time as the main problem [5]. With the standard guidelines set at a maximum reading rate of 10 frames per second in a single view mode, reading of capsule images has been associated with a significant burden on the clinician with a risk of interpretation errors because of the associated eye strain [9]. The European Society of Gastrointestinal Endoscopy, in a bid to reduce the workload and minimize errors, recommends, in part, pre‐reporting be done by nurses and technicians who are duly trained; however, this comes at an additional human resource cost [9].

Several software and tools have been developed in an attempt to reduce the reading time and increase accuracy, without which the ease of use, safety, diagnostic yield, and patient acceptability brought about by SBCE is limited [5]. The suspected blood indicator (SBI) tool, for instance, attempts to identify and tag possible hemorrhagic areas in the video frames with a red pixel for ease of identification. There is, however, a significant compromise in reading quality and validity with the utility of these reading aid tools such as the SBI with recommendations for adjunctive automated reading software and the use of artificial intelligence (AI). Machine learning (ML) models such as support vector machines developed for the detection of small bowel bleeding were initially limited by their difficulty in 2D image recognition. This has in part been addressed by the convolutional neural network (CNN), which utilizes deep learning (DL). The discovery of CNN greatly paved the way for a myriad of AI models in image classification beyond small bowel bleeding and into other lesions detection [7].

Almost two and a half decades later since the inception of SBCE and with several advancements including the introduction of AI, its effectiveness in small bowel diagnostics remains well appreciated. There are, however, still conflicting reports on the diagnostic accuracy, clinical validity, and effectiveness of different AI models in the diagnosis of small bowel lesions in comparison to conventional endoscopy. This systematic review and meta‐analysis provide a comprehensive report of this.

## 2 Methodology

### 2.1 Study Protocol and Registration

This systematic review and meta‐analysis were conducted in accordance with the Preferred Reporting Items for Systematic Review and Meta‐Analysis Protocols (PRISMA‐P) and Assessing the Methodological Quality of Systematic Reviews (AMSTAR) guidelines [10, 11]. The protocol for the study was registered in the International Prospective Register of Systematic Reviews (PROSPERO).

### 2.2 Data Sources and Search Strategy

Literature searches were performed through PubMed/MEDLINE, SCOPUS, and EMBASE to identify studies eligible for inclusion. All publications up to November 24, 2024, the latest search date, were included. Search terms used for the three databases included are shown in Table 1. No restrictions on language or study type were specified on the search protocol. The PubMed function “related articles” was used to extend the search to provide a reference list of all included studies. Backwards citation was used when appropriate to include pertinent articles. The following PICOS criteria were used as a framework to design the study question and formulate the literature search strategies to ensure comprehensive and bias‐free searches:

**TABLE 1:** Search strategy for the databases utilized in the study.

Database	Search strategy
PubMed	((AI OR “artificial intelligence” OR “machine learning” OR “deep learning” OR “neural network” OR “digital image analysis”) AND (“small bowel”) AND (“capsule endoscopy”) AND (“detection” OR “diagnosis” OR “diagnosing”))
SCOPUS	TITLE‐ABS‐KEY (((AI OR “artificial intelligence” OR “machine learning” OR “deep learning” OR “neural network” OR “digital image analysis”) AND (“small bowel”) AND (“capsule endoscopy”) AND (“detection” OR “diagnosis” OR “diagnosing”)))
EMBASE	((AI OR “artificial intelligence” OR “machine learning” OR “deep learning” OR “neural network” OR “digital image analysis”) AND (“small bowel”) AND (“capsule endoscopy”) AND (“detection” OR “diagnosis” OR “diagnosing”))

P (Population): adults (> 18) with small bowel lesions.

I (Intervention): AI‐assisted SBCE.

C (Comparison): Conventional SBCE.

O (Outcomes): Detection of small bowel tumors, Crohn's disease, polyposis syndrome, Celiac disease, and obscure intestinal bleeding.

S (Studies): Original articles (including observational studies, randomized control trials) systematic reviews, meta‐analyses, and case series.

### 2.3 Eligibility Criteria and Screening of Articles

Rayyan citation manager was used to facilitate screening of articles obtained from the search process. Duplicate citations were cross‐checked manually and removed after careful evaluation of the data. Title and abstract of the remaining articles were screened for relevance, and full texts were obtained for those that passed the inclusion criteria. For repeat articles from the same group containing a search period overlap and similar data sets, only the most recent article was included to avoid duplication of data.

Studies were considered eligible for inclusion if they contained relevant information on the use of AI‐assisted ML algorithms in SBCE for the detection of small bowel lesions in adult patients. The following criteria were used to establish eligibility of studies—inclusion criteria: original articles (including observational studies, case control, cohort studies, and randomized control trials) systematic reviews, meta‐analyses, and case series specific to AI‐assisted SBCE in adults; exclusion criteria: narrative reviews, editorials, short communications, opinion articles, case studies, and articles for which the full text was not retrievable and in cases where actual patients were not utilized in the study. Non‐English articles were excluded at this stage, as were studies with incomplete or irrelevant information. Two authors independently undertook the above, and any disagreements about eligibility were settled through consensus with a third reviewer. A PRISMA flow chart was developed to outline this.

### 2.4 Data Extraction and Outcomes of Interest

All relevant articles that passed the screening and inclusion criteria were considered for analysis. Data extraction was conducted by two independent reviewers and any consensus settled by a third reviewer. Data extraction was done using a standard excel sheet generated with the variables to be extracted from each of the studies included.

From each study, the following information was extracted: study characteristics—authors, original title, full article abstract, publication year, country and continent, study design, sample size, and study period; participant demographics—age, sex, and clinical characteristics (e.g., symptoms, risk factors, and comorbidities); intervention details—description of the AI‐assisted SBCE system (e.g., type of algorithm, training data) and the standard conventional SBCE procedures; outcome measures—diagnostic accuracy, sensitivity, specificity, diagnostic odds ratio, positive predictive value (PPV) and negative predictive value (NPV), mean procedure/reading time, small bowel transit time, all GI tract transit time, time to detect one small bowel lesion, complications, and interobserver agreement.

Our meta‐analysis aims to comprehensively evaluate the performance of AI‐assisted ce compared with conventional methods by human clinicians in the detection of small bowel lesions. The analysis aims to encompass multiple studies, covering a range of metrics including accuracy, sensitivity, specificity, PPV, and NPV.

### 2.5 Data Summary and Synthesis

The data were entered into an Excel sheet for cleaning, validation, and coding. The data were presented in a tabular form for presentation. The extracted data were pooled, and a meta‐analysis was performed for the appropriate variables, considering the clinical and methodological heterogeneity among the included studies. Comprehensive Meta Analysis v4.0 (Biostat Inc.) was used for the analysis of the data.

### 2.6 Meta‐Analysis of Diagnostic Test Accuracy

Meta‐analysis was conducted only on full‐text articles that provided complete descriptive statistical data, including confidence intervals. Pooled accuracy, sensitivity, specificity, PPV, and NPV were determined for AI‐assisted ce versus conventional endoscopy. Forest plots with a 95% CI were calculated and pooled, and pooled interval data were assessed. Heterogeneity among the outcomes of included studies in this meta‐analysis was evaluated using Cochrane's *Q* test. Significant heterogeneity was indicated by *p* less than 0.05 in Cochrane's *Q* test. For results with significant heterogeneity, a random‐effects model was utilized. And those with nonsignificant heterogeneity, a fixed‐effects model was performed. Statistical analyses were performed using Python programming language v3.4 (Python Software Foundation, Wilmington, Delaware). Data analysis and visualization were completed using Comprehensive Meta‐Analysis v4.0 (Biostat Inc.).

### 2.7 Risk of Bias Assessment

The quality of the included studies was assessed using the appropriate tools for each study design. For observational studies, the risk of bias assessment tool for nonrandomized studies (RoBANS II) was used, whereas the Cochrane Risk of Bias tool (ROB2) was employed for RCTs. Two independent reviewers assessed the quality of each study, with disagreements resolved through discussion or consultation with a third reviewer if necessary.

## 3 Results

### 3.1 Summary of Study Characteristics

**FIGURE 1:**
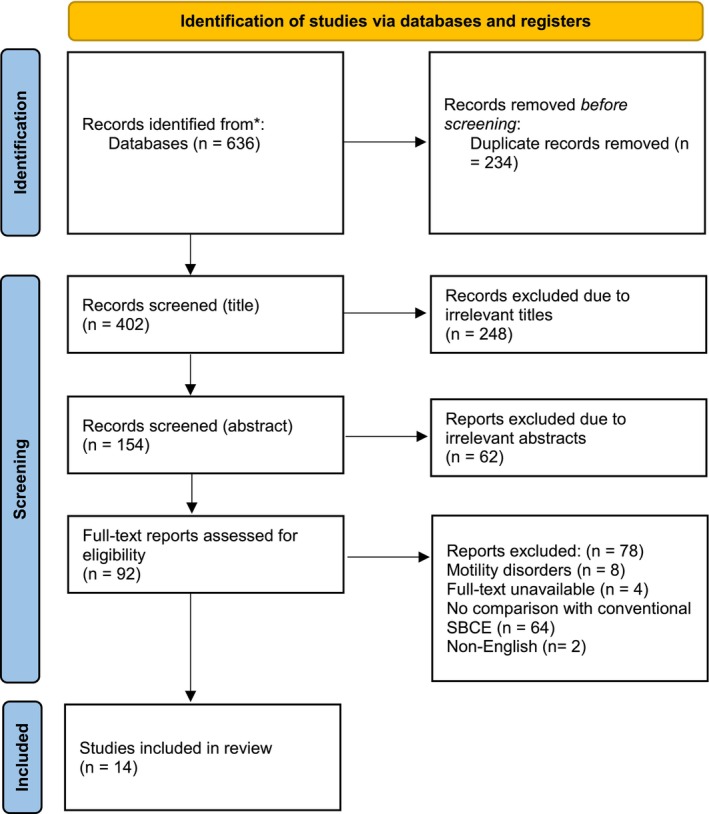
Preferred Reporting Items for Systematic Review and Meta‐Analysis (PRISMA) flowchart for included studies.

A total of 14 studies [12, 13, 14, 15, 16, 17, 18, 19, 20, 21, 22, 23, 24, 25] were included, and the breakdown for screening according to the PRISMA guidelines is as presented in Figure 1. A detailed summary of key findings from each study is as presented in Table 2.

**TABLE 2:** Comprehensive summary of the studies included in our review.

First author	Year	Study design	Sample size	AI algorithm	Accuracy	Sensitivity	Specificity
João Afonso [12]	2022	Retrospective	1229 patients, 1483 capsule endoscopy exams	Convolutional neural network (CNN): Xception model with its weights trained on ImageNet. Tensorflow 2.3 and Keras libraries to prepare the data and run the model.	95.60% (SD 2.18%)	90.82% (SD 4.68%)	93.41% (SD 3.32%)
Tomonori Aoki [13]	2020	Retrospective	20 SBCE videos	CNN—Single Shot MultiBox Detector	NA	NA	NA
Adriana Florentina Constantinescu [14]	2016	Prospective	54 patients	Artificial neural networks	NA	Human—94.79%; computer‐aided diagnosis/ANN—93.75% (*p* = 0.231)	Human—93.68%; computer‐aided diagnosis/ANN—91.38% (*p* = 0.231)
Zhen Ding [15]	2023	Retrospective	Training phase—2565 cases, 280 426 images; validation phase—240 videos	CNN‐based model Plus a convolutional recurrent neural network (CRNN) model	AI model 89.4% (CI 87.9–90.7); junior doctors 85.5% (83.9–87.1); experts 96.6% (95.7–97.4); junior doctor + AI assisted 97.9% (97.1–98.5)	AI model 96.5% (CI 94.9–97.7); junior doctors 65.91 (62.3–69.3); experts 91.1% (88.8–93.1); junior doctors + AI assisted 99.2% (98.2–99.7)	AI model 85.1% (CI 83.0–87.1); junior doctors 97.3% (96.2–98.1); experts 100.0% (99.7–100.0); junior doctors + AI assisted 97.14% (96.0–98.0)
Zhen Ding[16]	2019	Retrospective	6970 patients (113 426 569 images); 1970 cases were used in the training phase, and 5000 cases were used to validate the model	CNN‐based auxiliary reading model	NA	74.57% (95% CI, 73.05–76.03)—conventional reading; 99.88% (95% CI, 99.67–99.96)—CNN‐based auxiliary testing	100% (95% CI, 99.72–100)—conventional reading; 100% (95% CI, 99.72–100)—CNN‐based auxiliary testing
Antonio Giordano [17]	2023	Retrospective	111 SBce videos	TOP100, an integrated AI tool that selects the 100 most relevant frames, including potential lesions from SBce video recording.	TOP100 AI tool—92.79 (86.29–96.84).	TOP100 AI tool—90.48% (95% CI 82.09–95.80)	TOP100 AI tool—specificity of 100% (95% CI 87.23–100)
Jeongwoo Ju [18]	2023	Retrospective	100 patients, 300 video clips	DeepLab v3, whose backbone model was ResNet50	NA	NA	NA
Miguel Mascarenhas [25]	2024	Retrospective cohort	5846 CE exams in 4372 patients	CNN	97.6 (97.5–97.7)	78.6 (76.9–80.7)	97.6 (97.5–97.7)
Fintan John O'Hara [19]	2023	Retrospective	40 patients/procedures	Not specified	NA	AI‐assisted reading—98.1% (95% CI 97.15–98.7); standard reading mode—86.2% (95% CI 84.2–87.9), *p* < 0.001	NA
Miguel Mascarenhas Saraiva [20]	2021	Retrospective	1229 subjects; 1483 CE exams	CNN, Xception model with its weights trained on ImageNet	98.5% (95% CI 98.6–98.6)—CNN.	98.6% (95% CI 97.6–99.7)—CNN	98.9% (95% CI 97.6–99.7)—CNN
Cristiano Spada (P1 + P2 lesion) [24]	2024	Prospective trial	133 patients/procedures	Deep CNN	78.2% (70–85)	91.6 (83–97)	56 (41–70)
Cristiano Spada (P2 lesions) [24]	2024	Prospective trial	133 patients/procedures	Deep CNN	87.2 (80–92)	87.3 (76–95)	87.2 (78–94)
Xia Xie [21]	2022	Retrospective	5825 SBCE examinations in total; 2898 of the total examinations used for validation	CNN‐based CADe algorithm, SmartScan (trained and validated with the present study)	NA	98.8% (95% CI, 98.3%–99.2%) in SmartScan‐assisted reading; vs. 88.1% (95% CI, 86.7%–89.3%) in conventional reading; *p* < 0.001	NA
Stefania Chetcuti Zammit [22]	2023	Retrospective	63 VCE videos from 63 patients with biopsy confirmed celiac disease	CNN—ResNet 50 (with 64 3 × 3 filters at the lowest level, and four layers, each with a 3,4,6,3 residual block schedule. The architecture was implemented in Python v3.6 using ANTsPyNet and TensorFlow as the backend)	NA	NA	NA
Stefania Piccirelli [23]	2022	Prospective	126 patients	Express View (EV): a reading software based on conventional machine learning (ML) algorithm	Express View (AI) reading diagnostic accuracy 93% (CI 86.5–96.6); standard reading diagnostic accuracy 93% (CI 87.5–97.2)	Express View (AI) reading 88% (CI 79.2–94.6); standard reading 91% (CI 82.4–96.3)	Express View (AI) reading 100% (CI 90.5–100); standard reading 98% (CI 88–100)

### 3.2 Patient Demographics

The mean of reported ages for included studies was 54.3 years (SD 17.7); 55.4% of individuals were men, and 44.6% were women. Seven studies were conducted within Europe and five in Asia. The most commonly reported symptoms included gastrointestinal bleeding, abdominal pain, chronic diarrhea, and iron deficiency anemia. The most common comorbidity identified was Crohn's disease. The commonly utilized ML algorithm were CNNs including the Xception model, DeepLab v3, SmartScan, and ResNet 50, followed by a hybrid approach with CNN + convolutional recurrent neural network (CRNN) model. The use of artificial neural networks (ANN) was reported in one study. One study employed Express View (an AI system based on conventional ML algorithms), whereas another study utilized TOP100 (an integrated AI tool that selects the 100 most relevant frames, including potential lesions from SBCE video recordings) [17, 23]. Data regarding complications were reported only in two studies. Chetcuti Zammit et al. reported an incomplete examination (*n* = 17), delayed small bowel transit time (*n* = 11), small bowel stricture (*n* = 2), and delayed gastric transit time (*n* = 4) [22]. Constantinescu et al. reported no complications [14].

### 3.3 Transit and Reading Times

The average number of reported images/frames was 37 097 854 (range: 4904–148 357 922). The gold standard for comparison of AI outcomes was against consensus diagnosis from experts, which consisted of board‐certified gastroenterologists. The mean conventional reading time is estimated 43.9 min (SD 27.82), and the mean AI reading time is 5.7 min (SD 4.8). The mean reported frames per second for AI training of the image set was 106.7 frames per second (SD: 68.8). The pooled mean small bowel transit time was 312 min (SD: 54). Data regarding interobserver agreement were only provided by Ju et al. [17]. The five gastroenterologists evaluated ce clip video quality as “high” in 10.7%–36.7% and as “low” in 28.7%–60.3% and 29.7% of cases, respectively. The AI evaluated ce clip video quality as “high” in 27.7% and as “low” in 29.7% of cases. Bonferroni's multiple comparison tests showed no significant difference between three gastroenterologists and AI (*p* = 0.0961, *p* = 1.0000, and *p* = 0.0676, respectively) but a significant difference between the other 2 with AI (*p* < 0.0001).

### 3.4 Conventional ce—Pooled Accuracy

A total of two studies were included in the conventional accuracy analysis [11, 12]. A random‐effects model was employed for the analysis. The mean effect size is 0.966 with a 95% confidence interval of 0.925–0.988. Given that only two studies provided complete data that could have been pooled, an *I*‐squared statistic and heterogeneity analysis could not be completed.

### 3.5 AI ce—Pooled Accuracy

**FIGURE 2:**
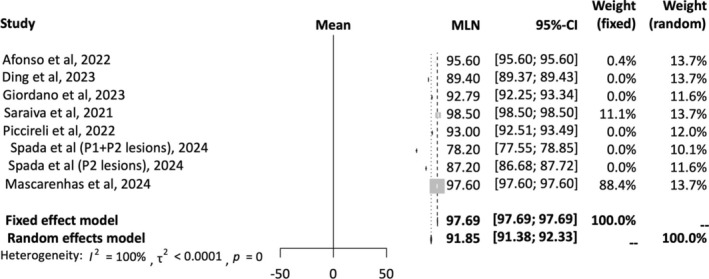
Pooled accuracy for AI‐assisted capsule endoscopy.

A total of eight studies were included in the AI accuracy analysis [12, 15, 17, 20, 23, 24]. A random‐effects model was employed for the analysis. The mean effect size is 91.85% with a 95% confidence interval of 91.38%–92.33%. The *I*‐squared statistic is 100%, which informs us that the observed effects reflect variance in true effects rather than sampling error. Figure 2 provides a forest plot depicting pooled accuracy of AI detection of SBce.

### 3.6 Conventional CE—Pooled Sensitivity

A total of five studies were included in the conventional sensitivity analysis [14, 17, 21, 22, 23]. A random‐effects model was employed for the analysis. The mean effect size is 0.860 with a 95% confidence interval of 0.786–0.934. The *Q*‐statistic provides a test of the null hypothesis that all studies in the analysis share a common effect size. The *Q*‐value is 240.979 with four degrees of freedom and *p* < 0.001. Using the criterion alpha, we can reject the null hypothesis that the true effect size is the same in all these studies. The *I*‐squared statistic is 98%, which informs us that 98% of the observed effects reflect variance in true effects rather than sampling error.

### 3.7 AI ce—Pooled Sensitivity

**FIGURE 3:**
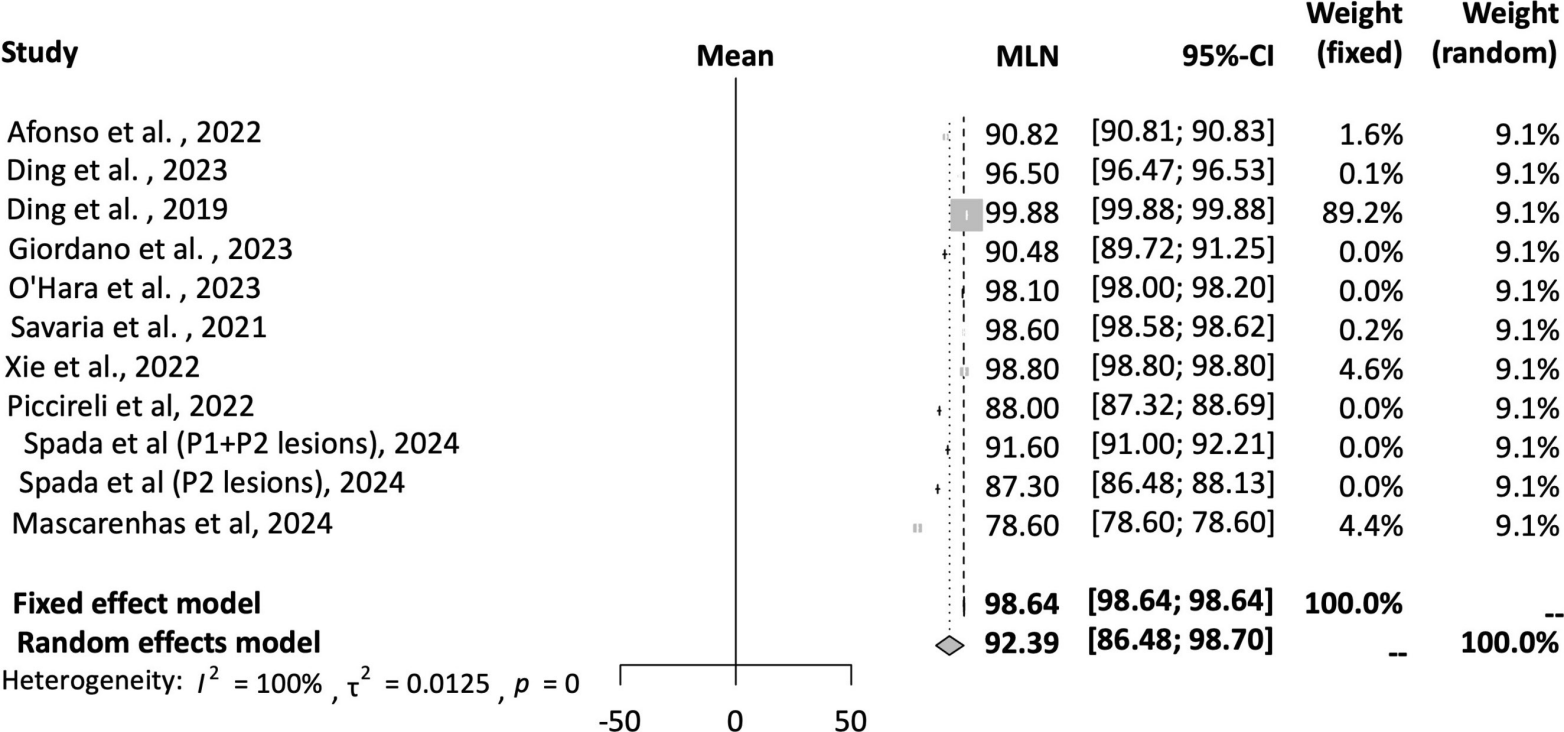
Pooled sensitivity for AI‐assisted capsule endoscopy.

A total of 11 studies were included in the AI sensitivity analysis [12, 15, 16, 17, 19, 20, 21, 23, 24, 25]. A random‐effects model was employed for this analysis. The mean effect size is 92.39% with a confidence interval of 86.48%–98.70%. The *I*‐squared statistic is 100%, which informs us that the observed effects reflect variance in true effects rather than sampling error. Figure 3 provides a forest plot depicting pooled sensitivity of AI detection of SBce.

### 3.8 Conventional CE—Pooled Specificity

A total of three studies were included in the conventional specificity analysis [15, 18, 22]. A random‐effects model was employed for the analysis. The mean effect size is 0.998 with a 95% confidence interval of 0.996–0.999. The *Q*‐statistic provides a test of the null hypothesis that all studies in the analysis share a common effect size. The *Q*‐value is 5.628 with two degrees of freedom and *p* = 0.060. Using the criterion alpha, we cannot reject the null hypothesis that the true effect size is the same in all these studies. The *I*‐squared statistic is 64%, which informs us that 64% of the observed effects reflect variance in true effects rather than sampling error.

### 3.9 AI ce—Pooled Specificity

**FIGURE 4:**
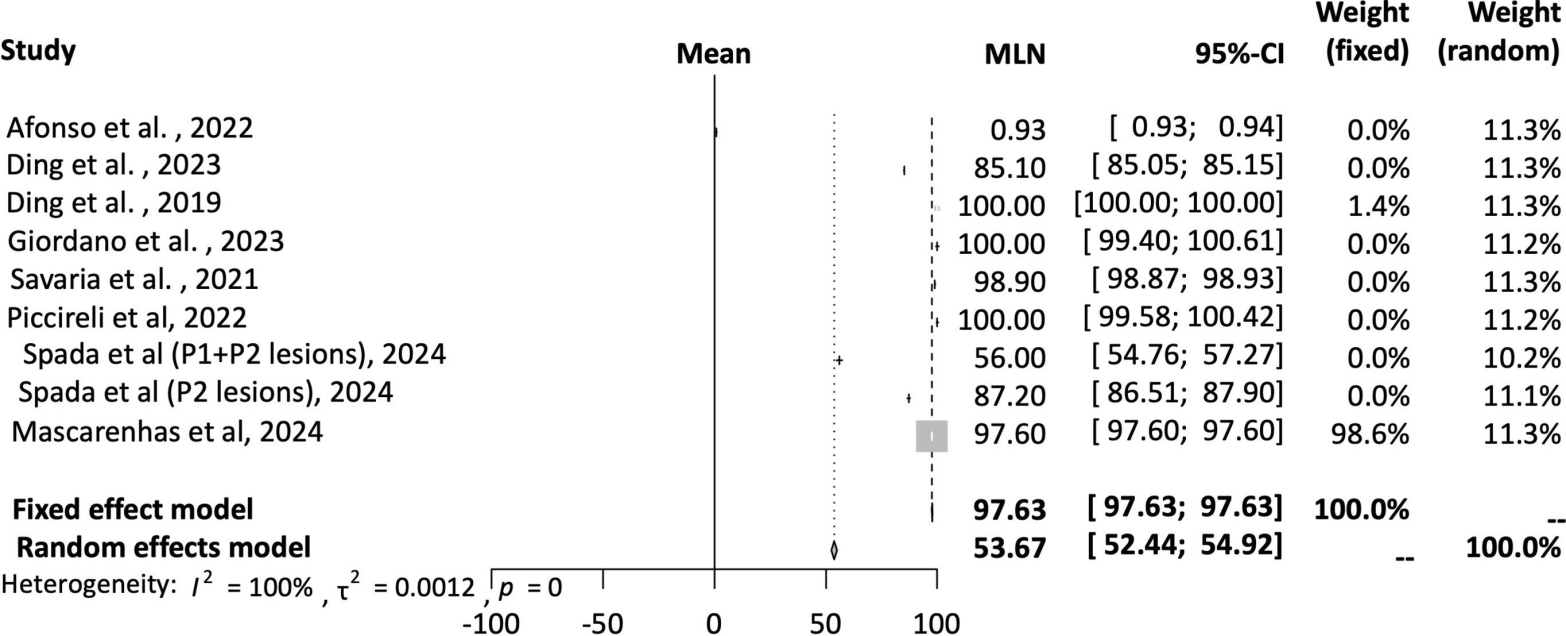
Pooled specificity for AI‐assisted capsule endoscopy.

A total of nine studies were included in the AI specificity analysis [12, 15, 16, 17, 20, 23, 24, 25]. A random‐effects model was employed for this analysis. The mean effect size is 53.67% with a confidence interval of 52.44%–54.92%. The *Z*‐value is 53.209 with *p* < 0.001. The *I*‐squared statistic is 100%, which informs us that the observed effects reflect variance in true effects rather than sampling error. Figure 4 provides a forest plot depicting pooled specificity of AI detection of SBce.

### 3.10 Conventional ce—Pooled PPV

A total of three studies were included in the conventional PPV analysis [15, 18, 22]. A random‐effects model was employed for this analysis. The mean effect size is 0.982 with a confidence interval of 0.976–0.987. The *Q*‐value is 0.180 with two degrees of freedom.

### 3.11 AI ce—Pooled PPV

**FIGURE 5:**
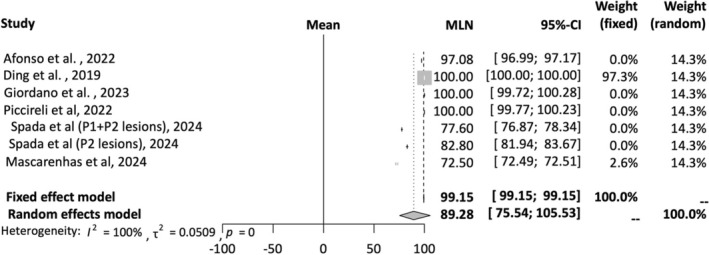
Pooled positive predictive value for AI‐assisted capsule endoscopy.

A total of seven studies were included in the AI PPV analysis [12, 16, 17, 23, 24, 25]. A random‐effects model was employed for this analysis. The mean effect size is 89.28% with a confidence interval of 75.54%–99.9%. The *I*‐squared statistic is 99%, which informs us that the observed effects reflect variance in true effects rather than sampling error. Figure 5 provides a forest plot depicting pooled PPV of AI detection of SBce.

### 3.12 Conventional CE—Pooled NPV

A total of two studies were included in the conventional NPV analysis [15, 18]. A random‐effects model was employed for this analysis. The mean effect size is 0.760 with a confidence interval of 0.577–0.943. Given that only two studies were available for this analysis, a reliable *Q*‐value or *I*‐squared statistic could not be calculated.

### 3.13 AI CE—Pooled NPV

**FIGURE 6:**
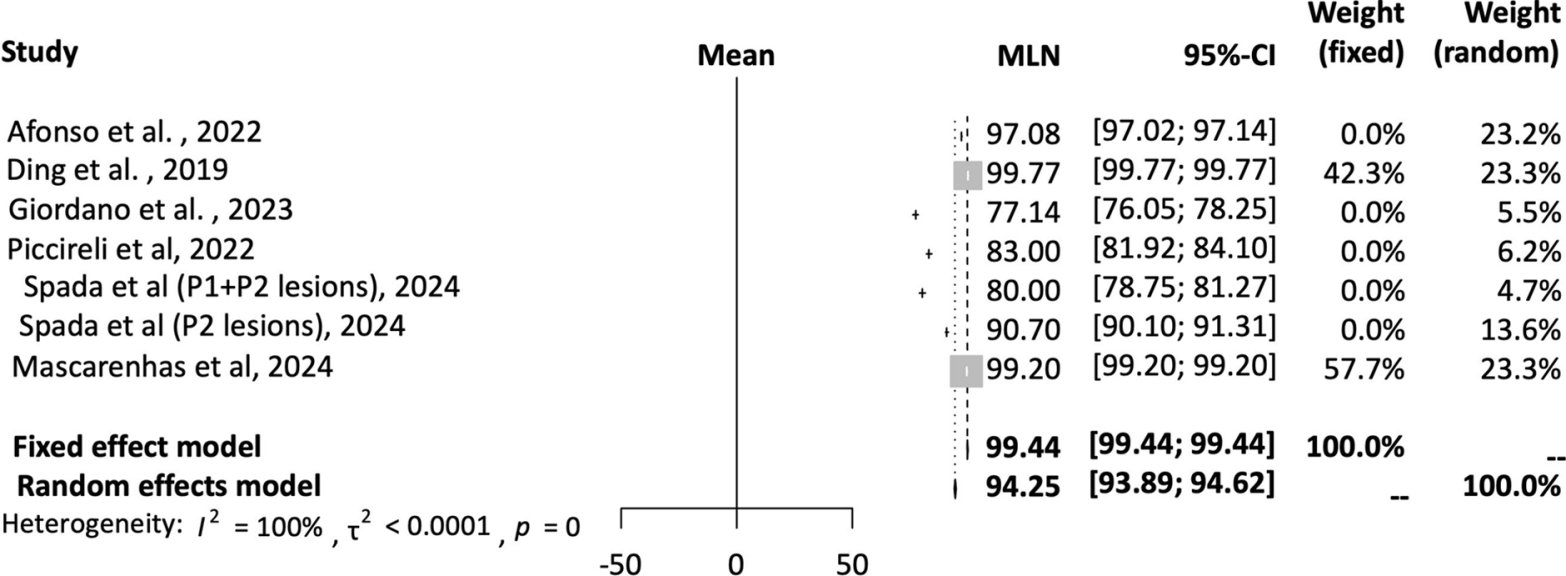
Pooled negative predictive value for AI‐assisted capsule endoscopy.

A total of seven studies were included in the AI NPV analysis [12, 16, 17, 23, 24, 25]. A random‐effects model was employed for this analysis. The mean effect size is 94.25 with a confidence interval of 93.89%–94.62%. The *I*‐squared statistic is 99%, which informs us that the observed effects reflect variance in true effects rather than sampling error. Figure 6 provides a forest plot depicting pooled NPV of AI detection of SBCE.

### 3.14 Quality and Risk of Bias Assessment

**FIGURE 7:**
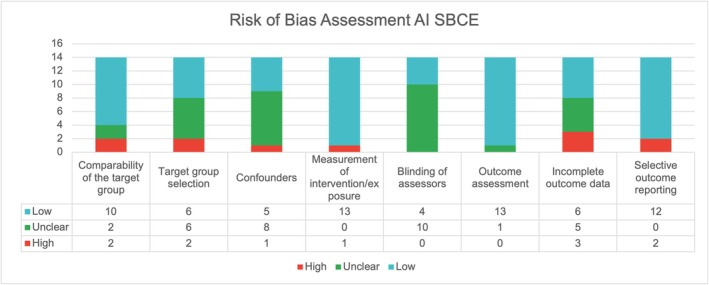
Risk of bias assessment using the RoBANS II tool.

We evaluated all nonrandomized studies using the RoBANS II tool [12, 13, 14, 15, 16, 17, 18, 19, 20, 21, 22, 23, 24, 25]. Figure 7 visually represents the risk of bias analysis for these trials. Generally, the risk of bias for the studies included in this systematic review was minimal. The domain noted to most likely have a high risk of bias pertained to incomplete outcome data, whereas the risk of bias for the domain “blinding of assessors” was often unclear. The domain “outcome assessment” exhibited the least bias.

## 4 Discussion

### 4.1 Medical Imaging and CNN Model

The incorporation of AI into medical imaging stands as a revolutionary advancement, akin in significance to the groundbreaking efforts of Roentgen, Becquerel, and Curie. In the field of medical imaging, ANN serves as the fundamental element for both ML and DL. An ANN is an analytical algorithm comprising interconnected layers of nodes, where inputs may include radiomic features from image files or, in the case of a CNN, the images directly. An ANN is identified by nodes ranging from hundreds to millions, arranged in layers, often referred to as depth [26]. DL utilizes an ANN with multiple layers, typically surpassing six, representing a more sophisticated iteration of ML. DL excels in intricate analyses, assimilating extensive data and representing elevated levels of abstraction.

For a CNN, it incorporates convolution and pooling layers to extract features from images, generating an output usually in some form of a classification [26]. ANNs rely on data and yield outcomes constrained by the quality of the input data. In the fields of radiology and nuclear medicine, a CNN can be fed an image or a series of images, whereas extracted radiomic features can serve as input for an ANN. Notably, a CNN is adept at autonomously identifying and extracting radiomic features from input images, linking them to outcomes to enhance overall results [27].

### 4.2 AI‐Guided ce

#### 4.2.1 Gastrointestinal Hemorrhage

ce is an effective initial diagnostic method for evaluating patients with occult gastrointestinal bleed with moderate evidence and strong recommendation [28]. GI bleeding detection is essential for ce examination in that bleeding is not only the most common abnormality of the GI tract but also an important symptom or syndrome of other GI pathologies such as ulcers, polyps, tumors, and Crohn's disease [29]. In 2016, Jia et al. developed a CNN model for detection of GI bleed and showed superior precision, recall and F1 scores compared with conventional handcrafted techniques for bleed detection [30].

#### 4.2.2 Erosion and Ulcers

Mucosal disruptions, such as erosions and ulcerations, represent the most prevalent abnormalities observed in the small bowel during ce. These anomalies are frequently attributed to the usage of nonsteroidal anti‐inflammatory drugs (NSAIDs) and, on occasion, to conditions like Crohn's disease or small‐bowel malignancy. Given this, timely diagnosis and intervention assume significant importance [30, 31].

Aoki et al. pioneered the development of a CNN‐based system designed for the automatic detection of ulcers and erosions in ce images [32]. Their model showcased robust overall performance, indicated by an AUROC of 0.958, coupled with a sensitivity of 88% and specificity of 91% [12]. In 2019, Klang and collaborators introduced a DL system achieving remarkably accurate detection of ulcers and erosions, with reported accuracy ranging from 95% to 97% [33].

#### 4.2.3 Angioectasia

Small‐bowel angioectasia is a collection of abnormal blood vessels composed of thin tortuous capillaries without an internal elastic membrane. Small‐bowel angioectasia comprises the majority of small‐bowel vascular lesions and is found in 30%–40% of obscure gastrointestinal bleeding (OGIB) cases [34]. The advancement of AI mechanisms for the automatic detection of gastrointestinal lesions has primarily concentrated on ce, particularly for identifying small bowel angioectasia. Numerous studies have specifically targeted the computer‐aided detection of vascular lesions in the small bowel using ce. Notably, OGIB is the predominant indication for both ce and device‐assisted enteroscopy (DAE), with vascular lesions, particularly angioectasia, being the most prevalent etiology.

Pioneering this area, Noya and colleagues conducted the first study describing the outcomes of an AI algorithm developed for the automatic detection of small bowel angioectasia [35]. Their model demonstrated a sensitivity of 90%, specificity of 97%, and an AUROC of 0.93. In 2019, Leenhardt et al. presented a CNN capable of detecting angioectasia with a sensitivity of 100% and specificity of 96% [36]. These findings were subsequently corroborated by Tsuboi et al., who introduced a DL system with a high diagnostic yield for angioectasia detection, showcasing a sensitivity of 98.8% and specificity of 98.4% [37].

#### 4.2.4 Polyps and Tumors

The utilization of AI‐assisted CE presents a significant advancement in the detection of polyps and tumors within the small bowel [38]. This superior performance could potentially revolutionize early detection and intervention, crucial in improving patient outcomes, especially in cases of precancerous lesions or early‐stage tumors.

Yuan et al. pioneered a novel deep feature learning method named stacked sparse autoencoder with image manifold constraint (SSAEIM) to recognize polyps in the capsule endoscope images. They achieved an overall recognition accuracy of polyps of 98% while subclassifying normal images as either turbid, bubble, or clear [39]. Saito et al. further developed and tested a novel deep CNN‐based model for detecting protruding lesions using 93 patients and 17 507 images, having an overall sensitivity and specificity of 90.7% and 79.8%, respectively. This CNN‐based model further categorized lesions as either polyp, nodules, epithelial tumors, submucosal tumors, and venous structures with 85.6%, 92.0%, 95.8%, 77.0%, and 94.4% sensitivities, respectively [40].

Ongoing research aims to refine AI algorithms to enhance sensitivity and specificity in detecting lesions, improving the overall accuracy and reliability of CE for diagnosis. As AI algorithms continue to evolve and improve, AI‐assisted CE may become a standard tool in gastroenterology for screening, diagnosis, and monitoring of gastrointestinal conditions.

#### 4.2.5 Celiac Disease

Celiac disease diagnosis often necessitates detailed examination of the small bowel for characteristic lesions. Our analysis demonstrates the commendable performance of AI‐assisted CE, showcasing its high sensitivity (0.989) and specificity (0.951) in detecting lesions of the small bowel. The heightened accuracy compared with conventional endoscopy (pooled accuracy: 0.956) underscores the potential of AI in enhancing diagnostic accuracy and ensuring early identification of SBL.

Various DL modules have shown great potential in the accurate diagnosis of celiac disease. Wang et al. proposed a novel DL recalibration module with an accuracy, sensitivity, and specificity of 95.94%, 97.2%, and 95.63%, respectively [41]. Zhou et al. demonstrated that CNN‐based DL model achieved a 100% sensitivity and specificity in detection of celiac disease. Additionally, the detection potential of AI‐based ce was correlated with the disease severity index [42]. Consequently, the use of AI‐assisted ce can be used as a screening tool for patients with small bowel mucosal lesions to establish need for further testing such as biopsy and evaluate the severity of mucosal atrophy. This study, however, utilized a small sample size of 11 cases and 10 controls, necessitating further studies in this population.

Limitations to quality of output may be brought about by the effect of dark or extraneous substances. However, these may be improved by color masking, which results in improved distinction of celiac disease with villous atrophy [43]. Thus, the integration of AI into CE enhances diagnostic accuracy, expedites the identification of celiac disease, and ultimately supports timely intervention and management strategies for patients.

#### 4.2.6 Multiple Lesion Detection

One of the remarkable strengths of AI‐assisted CE lies in its capability to detect multiple lesions within the small bowel. AI algorithms meticulously analyze the vast amount of visual data captured by miniature cameras, swiftly identifying and characterizing anomalies such as ulcers, polyps, bleeding sites, or inflammatory lesions that may otherwise elude detection. CNN, a DL‐based method, employs a pattern recognition analytic model in three steps: feature extraction, feature dimension reduction, and final classification to identify multiple lesions [44].

CNN‐based auxiliary model identifies and characterizes bowel lesions with a higher sensitivity of 99.90% and a significantly reduced reading time of 5.9 min compared with conventional analysis by gastroenterologists of 96.6 min [15]. With this model, Ding et al. categorized 158 235 small bowel‐capsule endoscopic images from 1970 patients as either normal, inflammation, ulcer, polyp, lymphangiectasia, bleeding, vascular disease, protruding lesion, lymphatic follicular hyperplasia, diverticulum, parasite, or other [16]. This innovative approach marks a pivotal stride in gastrointestinal healthcare, empowering medical professionals to provide more comprehensive and targeted patient care.

#### 4.2.7 Capsule Localization

Accurate localization of lesions is pivotal for targeted interventions. Conventionally, indirect estimation of transit time after the duodenum to the cecum versus length of bowel and time of image capture has been employed to try and estimate the location of a lesion. This, however, has limited accuracy and more automated AI‐assisted methods are required for accurate bowel segmentation [45, 46]. In vitro studies using an unaltered capsule in an artificial bowel showed a mean error of less than 0.01 in 20 cm of travel, showing the potential for this technology. Introduction of additional sensors such as dual cameras, gyroscope, accelerometer, and magnetometer hold the hope for better capsule localization [44].

The structural modification incorporating additional sensors will ensure commendable accuracy in lesion localization, contributing to precise anatomical mapping within the small bowel. This improved localization, coupled with the high sensitivity and specificity of AI‐guided detection, underscores its potential in facilitating targeted therapies and minimizing unnecessary interventions.

### 4.3 Evaluating Clinical Performance of an Algorithm

Evaluation of the clinical performance of AI‐assisted models in the detection of small bowel lesions following SBCE provides insight into its overall diagnostic efficiency and application in clinical practice. Studies have sought to evaluate the utility of AI models and provide a standard model of assessment through the following parameters [44, 47]. These include diagnostic performance, clinician perception, application in health institutions, older versus newer models, source of data on its use, and its influence on treatment and monitoring [48]. The diagnostic performance of an algorithm is evaluated through sensitivity and specificity in the detection of SB abnormal images [5], diagnostic accuracy, capacity to reduce workload through increased time efficiency and high detection rate [16], and a unified diagnostic standard in the face of multiple DL models [7]. With the availability of various neural networks, the hallmark of an effective diagnostic algorithm is in a standardized method of diagnosis, a large database with multiple datasets to improve ML, and the prioritization of patient privacy and confidentiality, as AI‐led diagnosis in health institutions utilizes third party sources.

The effectiveness of an algorithm may also be influenced by the clinician perception and data sources. It has been seen that more established clinicians are less likely to adopt an AI diagnostic method than younger clinicians because of concerns about diagnostic accuracy and unified diagnostic standards [48]. In addition, multiple studies are done in the private setting and thus leave out crucial information that may be acquired in the public hospital environment, or in other specialized care centers and thus may not reflect patients in real‐world practice [44]. An algorithm's influence on disease treatment and monitoring is also crucial. In the detection of small bowel lesions, an advantage of AI models is the ability to transform purely qualitative and clinical observations into quantifiable and reproducible results [47]. This simplifies disease staging, may be applied in the calculation of therapeutic doses, and helps monitor disease progression.

### 4.4 Future Directions

This study illustrates the relatively high sensitivity and specificity of AI in diagnosing small bowel lesions through CE, as well as its numerous benefits over conventional diagnostic methods [16]. This field, however, is still in its infancy but holds a lot of promise for its future use in widespread clinical practice. A significant challenge in the AI‐assisted diagnosis is the lack of a streamlined and unified diagnostic standard across the different ML programs [44]. With ever‐increasing data, ML can be enhanced, and a unified database can be established, further strengthening diagnostic capacity.

In addition, there are continuous advances in DL software that enable the use of ML as a diagnostic tool. Therefore, there should be continuous prospective future studies investigating the progression of diagnostic efficiency of AI‐assisted diagnosis in SBCE, as it would improve clinician perception, enhance awareness, and increase funding to help advance this field. In addition, efforts to create available datasets on the effectiveness of models should be carried out in order to provide information on different models and their effectiveness, influence policy making, and enable reviews and updates to this diagnostic field. Studies should also include the ethical aspect of how well these models protect patient privacy, as this is an increasingly controversial topic in the healthcare industry that influences application in health institutions.

### 4.5 Limitations of This Study

The limitations of this study are that most data were obtained from retrospective studies. With numerous research projects still underway, diagnostic efficiency may change because of newer models and thus undermine this study's relevance. Multiple prospective studies must therefore be conducted to keep up with this ever‐growing field. In addition, publication bias must be considered, as factors such as the region of publication and clinician or investigator bias may affect result validity and subsequently the overall findings of our research. Studies that did not meet the inclusion criteria but contained key information may also have been overlooked.

## 5 Conclusion

AI‐assisted ce displays superior diagnostic accuracy, sensitivity, and PPVs albeit the lower pooled specificity in comparison with conventional ce. By leveraging advanced computational algorithms, AI enables clinicians to achieve a prompt and precise diagnosis of various small bowel pathologies. Its use would ensure accurate detection of small bowel lesions and further enhance their management.

The present meta‐analysis is constrained because of the limited number of studies incorporated. Prospective research, encompassing high‐caliber RCTs and the integration of AI‐aided ce into clinical settings, holds the promise of pioneering advancements in early identification, tailored therapeutic approaches, and enhanced prognostic assessments of small bowel disorders.

## Ethics Statement

The authors have nothing to report.

## Conflicts of Interest

The authors declare no conflicts of interest.

## Consent

The authors have nothing to report.

## Research Registration Unique Identifying Number (UIN)

The protocol for the study was registered in the International Prospective Register of Systematic Reviews (PROSPERO).
